# Supplementary material for: Device-Measured Physical Activity in 3,506 Individuals with Knee or Hip Arthroplasty
Source: Med Sci Sports Exerc. Author manuscript; Available in PMC 2024 May 1. (PMC7615832; doi:10.1249/MSS.0000000000003365)
Supplement: Supplemental Data File (.doc,.tif, pdf, etc.) [file EMS192558-supplement-Supplemental_Data_File___doc___tif__pdf__etc__.docx]

**Supplemental Digital Content**

eNote 1: UK Biobank Fields ii

eNote 2: ICD-9 and ICD-10 Codes iii

eTable 1: OPCS-4 Standalone Codes of Operation and Location iv

eTable 2: OPCS-4 Combination Codes of Operation and Location v

eTable 3: Unadjusted overall physical activity and mobility metrics by clinical cohort vi

eTable 4: Linear regression adjusted differences in additional machine learning behavioural classifications between non-arthritic participants and participants with any combination of hip and/or knee arthroplasty vii

eTable 5: Linear regression adjusted differences in additional machine learning behavioural classifications between non-arthritic participants and clinically-defined unilateral hip and knee participant cohorts viii

**eNote1: UK Biobank Fields**

31 – Sex

34 – Year of Birth

52 – Month of Birth

189 – Townsend Deprivation Index at Recruitment

1558 – Alcohol Intake Frequency

3414 – Hip pain for 3+ months

3773 – Knee pain for 3+ months

6138 – Qualifications

20116 – Smoking Status

21000 – Ethnic Background

23101 – Body Mass Index

40046 – Sleep – Overall Average

40047 – Sedentary – Overall Average

40048 – Light – Overall average

40049 – Moderate-Vigorous – Overall Average

90001 - Accelerometer Data .cwa format (bulk)

90011 – End time of wear (accelerometer),

90015 – Data quality, good wear time (accelerometer)

90016 – Data quality good calibration (accelerometer)

**eNote 2: ICD9/10 Codes for Osteoarthritis (Hospital Episode Statistics)**

ICD-9 and ICD-10 diagnostic codes were used to identify diagnoses of hip or knee osteoarthritis at any time point. ICD-9 codes for hip and knee osteoarthritis included 71515 and 71516. ICD-10 codes for diagnosed knee arthritis included M170, M171, M172, M173, M174, M175, M179, and associated sublevels. ICD-10 codes for diagnosed hip arthritis included M160, M161, M162, M163, M164, M165, M166, M167, M169 and associated sublevels.

| eTable 1: OPCS-4 Standalone Codes of Operation and Location | | | |
| --- | --- | --- | --- |
| Primary Knee Arthroplasty | Revision Knee  Arthroplasty | Primary Hip Arthroplasty | Revision Hip Arthroplasty |
| O181 | O180 | W371 | W370 |
| O188 | O182 | W378 | W372 |
| O189 | O183 | W379 | W373 |
| W401 | O184 | W381 | W374 |
| W408 | W400 | W388 | W380 |
| W409 | W402 | W389 | W382 |
| W411 | W403 | W391 | W383 |
| W418 | W404 | W398 | W384 |
| W421 | W410 | W399 | W392 |
| W428 | W412 | W931 | W393 |
| W429 | W413 | W938 | W395 |
|  | W414 | W939 | W462 |
|  | W420 | W941 | W472 |
|  | W422 | W948 | W482 |
|  | W432 | W949 | W932 |
|  | W425 | W951 | W933 |
|  |  | W958 | W940 |
|  |  | W959 | W942 |
|  |  |  | W943 |
|  |  |  | W952 |
|  |  |  | W953 |
|  |  |  | W954 |

| eTable 2: OPCS-4 Combination Codes of Operation and Location | | | |
| --- | --- | --- | --- |
| Primary Knee Arthroplasty | Revision Knee  Arthroplasty | Primary Hip  Arthroplasty | Revision Hip Arthroplasty |
| W521 (with: Z846, Z765, Z845, Z844, Z774, or Z787) | W424  (with: Y032 or Y037) | W521, W531, W541, or W581 (with: Z843, Z761, or Z756) | W394  (with: Y032 or Y037) |
|  | W522, W523, W532, W533, W542, W543, W544, W553, W564, W574, W582, W603, W613, W641, or W642  (with: Z846, Z765, Z845, Z844, Z774, or Z787) |  | W522, W523, W533, W542, W543, W544, W572, W574, W582 (with: Z843, Z761, or Z756) |

| *eTable 3: Unadjusted overall physical activity and mobility metrics by clinical cohort* | | | | | | | |
| --- | --- | --- | --- | --- | --- | --- | --- |
| Activity Metric | Any Hip or Knee Arthroplasty^a^ | | Single Knee Arthroplasty^b^ | | Single Hip Arthroplasty^b^ | | Non-Arthritic Cohort  (n=68,389) |
|  | End-Stage Osteoarthritis^c^ (n=514) | 12+ Months Postop^d^  (n=2,992) | End-Stage Osteoarthritis^c^  (n=149) | 12+ Months Postop^d^  (n=566) | End-Stage Osteoarthritis^c^  (n=177) | 12+ Months Postop^d^  (n=843) |  |
| Step Count (steps/day) | 7,525 [5,236 – 9,775] | 7,947  [5,725 – 10,533] | 7,437 [5,235 – 9,240] | 8,035  [5,616 – 10,487] | 7,984  [5,645 – 10,124] | 8,712  [6,446 – 11,219] | 9,207  [6,987 – 11,814] |
| Peak Cadence (steps/min) | 106 [98 – 113] | 107 [99 – 114] | 103 [96 – 109] | 106  [98 – 112] | 108  [101 – 116] | 110  [102 – 117] | 113  [106 – 120] |
| Acceleration (m*g*) | 24.7 [20.4 – 29.5] | 24.6 [20.5 – 29.7] | 24.4  [19.2 – 27.7] | 24.0 [20.1 – 28.9] | 25.1  [21.6 – 30.2] | 25.4  [21.2 – 30.3] | 27.4  [22.8 – 32.9] |
| MVPA (min/day) | 20.1 [7.9 – 43.1] | 22.8 [8.8 – 44.6] | 23.6  [8.7 – 39.6] | 19.5 [8.0 – 40.1] | 20.1 [10.6 – 49.5] | 30.6  [12.3 – 53.1] | 34.9  [17.6 – 59.2] |
| Light Activity (hours/day) | 4.9 [4.0 – 6.0] | 4.9 [3.9 – 6.0] | 4.8  [3.7 – 5.9] | 4.9  [3.8 – 6.0] | 4.9  [4.1 – 5.9] | 4.9  [4.0 – 6.0] | 4.9  [3.9 – 6.1] |
| Sedentary Behaviour (hours/day) | 9.5 [8.4 – 10.7] | 9.5 [8.3 – 10.8] | 9.4  [8.4 – 10.8] | 9.5  [8.2 – 10.7] | 9.6  [8.6 – 10.4] | 9.4  [8.2 – 10.6] | 9.4  [8.2 – 10.6] |
| Sleep (hours/day) | 8.8 [8.1 – 9.6] | 8.8 [8.1 – 9.6] | 8.8  [8.1 – 9.7] | 8.9  [8.2 – 9.7] | 8.7 [8.1 – 9.5] | 8.8  [8.0 – 9.6] | 8.7  [8.1 – 9.5] |
| *Activity metrics reported as unadjusted median [interquartile range]*  *^a^Includes all participants with any combination of primary or revision knee or hip arthroplasties, excluding those who had their first arthroplasty less than 12 months before accelerometer wear (early recoverers), or more than 12 months after accelerometer wear (future first arthroplasty).*  *^b^Single Hip and Single Knee Arthroplasty Cohorts are subsets of the Any Hip or Knee Arthroplasties Cohort as defined in Figure 1*  ^c^*End-stage arthritis is considered having their first arthroplasty within 12 months after accelerometer wear*  ^d^*Accelerometer wear more than 12 months after the participant’s first primary knee or hip arthroplasty* | | | | | | | |

| *eTable 4: Linear regression adjusted differences in additional machine learning behavioural classifications between non-arthritic participants and participants with any combination of hip and/or knee arthroplasty* | | | |
| --- | --- | --- | --- |
| Metric | Reference Cohort | End-Stage Knee  and/or Hip Osteoarthritis  (n=514) | 12+ Months After  First Knee or Hip Arthroplasty  (n=2,992) |
| Light Activity (min/day) | Non-Arthritic  (n=68,389) | 5.7 [-2.4, 13.8]  *(p=0.170)* | 5.6 [2.1, 9.1]  *(p=0.002)* |
|  | End-Stage Knee  and/or Hip Osteoarthritis  (n=514) |  | -0.1 [-8.8, 8.6]  (p=0.987) |
| Sedentary Behaviour (min/day) | Non-Arthritic  (n=68,389) | 0.9 [-8.1, -9.9]  *(p=0.847)* | 1. [-2.9, 4.9]  *(p=0.619)* |
|  | End-Stage Knee  and/or Hip Osteoarthritis  (n=514) |  | 0.1 [-9.6, 9.8]  *(p=0.984)* |
| Sleep Time (min/day) | Non-Arthritic  (n=68,389) | -0.7 [-7.1, 5.6]  *(p=0.821)* | -3.7 [-6.5, -1.0]  *(p=0.007)* |
|  | End-Stage Knee  and/or Hip Osteoarthritis  (n=514) |  | -3.0 [-9.8, 3.8]  *(p=0.388)* |
| *Estimate [95% confidence interval] in activity metrics adjusted for age, sex, body mass index, season, Charlson comorbidity index, education, ethnicity, alcohol consumption, and smoking status. A negative difference indicates lower activity in the cohort of interest relative to the reference cohort.* | | | |

| *eTable 5: Linear regression adjusted differences in additional machine learning behavioural classifications between non-arthritic participants and clinically-defined unilateral hip and knee participant cohorts* | | | | | |
| --- | --- | --- | --- | --- | --- |
| Metric | Reference Cohort | End-Stage Knee  Osteoarthritis (n=149) | 12+ Months After Knee Arthroplasty (n=566) | End-Stage Hip  Osteoarthritis (n=177) | 12+ Months After Hip Arthroplasty (n=843) |
| Light Activity (min/day) | Non-Arthritic (n=68,389) | -0.4 [-15.4, 14.6]  (p=0.958) | 6.2 [-1.6, 13.9]  (p=0.119) | 3.5 [-10.1, 17.4]  (p=0.614) | -0.1 [-6.5, 6.2]  (p=0.967) |
|  | End-Stage Knee Osteoarthritis (n=149) |  | 6.6 [-10.2, 23.4]  (p=0.443) | 4.1 [-16.2, 24.4]  (p=0.694) | 0.3 [-15.9, 16.5] (p=0.974) |
|  | 12+ Months Post  Knee Arthroplasty (n=566) |  |  | -2.5 [-18.2, 13.2]  (p=0.755) | -6.3 [-16.2, 3.6]  (p=0.213) |
|  | End-Stage Hip Osteoarthritis (n=177) |  |  |  | -3.8 [-18.9, 11.3]  (p=0.621) |
| Sedentary Behaviour (min/day) | Non-Arthritic (n=68,389) | 4.2 [-12.5, 20.8]  (p=0.624) | -4.7 [-13.3, 3.0]  (p=0.287) | 3.3 [-12.0, 18.5]  (p=0.672) | 3.0 [-4.1, 10.0]  (p=0.407) |
|  | End-Stage Knee Osteoarthritis (n=149) |  | -8.8 [-27.5, 9.8]  (p=0.353) | -0.9 [-23.4, 21.6]  (p=0.940) | -1.2 [-19.2, 16.8]  (p=0.898) |
|  | 12+ Months Post  Knee Arthroplasty (n=566) |  |  | 8.0 [-9.5, 25.4]  (p=0.371) | 7.7 [-3.4, 18.7]  (p=0.173) |
|  | End-Stage Hip Osteoarthritis (n=177) |  |  |  | -0.3 [-17.1, 16.4]  (p=0.971) |
| Sleep Time (min/day) | Non-Arthritic (n=68,389) | 3.0 [-8.7, 14.7]  (p=0.615) | 1.4 [-4.6, 7.5]  (p=0.645) | -2.1 [-12.8, 8.7]  (p=0.708) | -3.0 [-8.0, 1.9]  (p=0.231) |
|  | End-Stage Knee Osteoarthritis (n=149) |  | -1.6 [-14.4, 11.5]  (p=0.813) | -5.1 [-20.9, 10.8]  (p=0.532) | -6.0 [-18.7, 6.6]  (p=0.350) |
|  | 12+ Months Post  Knee Arthroplasty (n=566) |  |  | -3.5 [-15.8, 8.8]  (p=0.579) | -4.5 [-12.2, 3.3] (p=0.260) |
|  | End-Stage Hip Osteoarthritis (n=177) |  |  |  | -1.0 [-12.8, 10.8]  (p=0.871) |
| *Difference [95% confidence interval] in activity metrics adjusted for age, sex, body mass index, season, Charlson comorbidity index, education, ethnicity, alcohol consumption, and smoking status. A negative difference indicates lower activity in the cohort of interest relative to the reference cohort.* | | | | | |
